# Examining Statewide Opioid Prescribing Limits and Prescription Drug Monitoring Program Mandates: Provider Compliance and Patient Outcomes

**DOI:** 10.1016/j.focus.2025.100460

**Published:** 2025-10-27

**Authors:** Jason Hoppe, Heather Tolle, Katherine J. Sullivan, Katherine Ziegler, Zachary Giano, Barbara Gabella

**Affiliations:** 1 Department of Emergency Medicine, University of Colorado School of Medicine, Aurora, Colorado; 2 Colorado Department of Public Health and Environment (CDPHE), Denver, Colorado; 3 Avera Research Institute, Sioux Falls, South Dakota; 4 Cross Appointment in the Department of Pediatrics and Department of Internal Medicine, Sanford School of Medicine, University of South Dakota, Vermillion, South Dakota; 5 Colorado School of Public Health, University of Colorado Anschutz Medical Campus, Aurora, Colorado

**Keywords:** Prescription drug monitoring program, opioid analgesics, health policy analysis, opioid use disorder, controlled substances

## Abstract

**Introduction:**

In response to the opioid crisis, Colorado enacted Senate Bill 18-022, which limits initial opioid prescriptions to 7 days for opioid-naïve patients and mandates prescription drug monitoring program review before a second prescription. This study evaluates provider adherence to these mandates and associated patient outcomes.

**Methods:**

A retrospective cohort study was conducted using linked electronic health record and prescription drug monitoring program data from a large academic-affiliated healthcare system in Colorado. Adult opioid-naïve patients (aged 18–89 years) who received an initial opioid prescription between May 2018 and May 2019 were included. Provider adherence to the 7-day prescribing limit for the first and second prescriptions and prescription drug monitoring program review mandate with the second opioid prescription were assessed. Patient outcomes included chronic opioid use, aberrant opioid use, and healthcare utilization within 6 months after the prescription.

**Results:**

Among 35,461 opioid-naïve patients prescribed an opioid, 78.5% received an initial prescription consistent with the 7-day statutory limit. Of the 9,423 patients with a second opioid prescription, 58.4% received a 7-day supply or less, and 13.1% had a documented prescription drug monitoring program review. Prescriptions exceeding 7 days were significantly associated with increased chronic opioid use (31.9% vs 0.2%), aberrant use (4.0% vs 2.1%), and higher healthcare utilization (mean 5.44 vs 1.95 visits). Prescription drug monitoring program review was not associated with reduced chronic opioid use or healthcare visits and was more common among patients with worse outcomes, suggesting potential confounding by patient complexity.

**Conclusions:**

Providers largely adhered to the 7-day opioid prescribing limit, which was associated with improved patient outcomes. However, mandated prescription drug monitoring program checks were infrequent and not associated with reduced risk, highlighting the need to reassess the effectiveness of prescription drug monitoring program–related policies.

## Highlights

• High adherence: Providers complied with statutory 7-day opioid prescribing limit in most cases.
• Mandated prescription drug monitoring program (PDMP) review occurred in only 13.1% of second opioid prescriptions.
• Opioid prescriptions over 7 days were linked to higher rates of chronic opioid use.
• PDMP review was associated with chronic opioid use and healthcare utilization.
• Novel linkage of PDMP and routinely collected electronic health records to assess outcomes.

## INTRODUCTION

Safe opioid analgesic prescribing is critical to effectively managing pain while minimizing unnecessary opioid exposure, preventing chronic opioid use, and reducing the pharmaceutical opioid supply contributing to the U.S. opioid crisis.^1^, ^2^, ^3^ In 2023, 8.6 million people misused opioids,^4^ and 75.4% of drug overdose deaths included an opioid, resulting in 81,083 lives lost.^5^ Although a majority of opioid deaths are now due to illicit opioid use, prescription opioids continue to be an important source of opioid exposures and contribute to overdoses. Evidence linking opioid analgesic use to long-term opioid use and misuse has driven policy interventions aimed at curbing overprescribing. These include mandates for use of prescription drug monitoring programs (PDMPs)^6^—state-wide databases containing dispensed controlled medications—and regulations that limit opioid prescribing or dispensing.^7^, ^8^, ^9^ PDMPs’ impact on prescribing practices, patient outcomes, and adverse events have yielded mixed results, raising concerns about unintended consequences such as undertreated pain, low quality care, and substitution with illicit opioids.^10^, ^11^, ^12^ Policy evaluations aimed at limiting opioids have shown reductions in the average days’ supply of opioids prescribed,^13^ the number of prescriptions exceeding a 7-days’ supply,^14^ and overall prescription volume.^15^ However, these population-level studies rarely assess patient-level outcomes, leaving gaps in understanding the impact on individuals. Healthcare legislation can increase administrative burdens on providers and divert attention from patient care, highlighting the need to investigate both intended and unintended consequences of regulatory policies.^16^

Evaluating state-level policies is essential to understand their potential benefits, unintended consequences, and the return on investment. Colorado’s statute, revised through Senate Bill 18-022 (SB18-022) in May 2018, aimed to improve patient safety by (1) limiting the first opioid analgesic prescription to ≤7-days’ supply for patients who had not received an opioid prescription from the same provider in the previous 12 months and (2) mandating that providers review the PDMP before issuing a second opioid prescription, (3) which was also limited to ≤7 days. Exceptions are allowed when pain is expected to last >90 days, pain is cancer-related pain, postsurgical pain is expected to last >14 days, and palliative or hospice care is provided.^17^

The objective of this study is to apply a novel method for linking visit-level PDMP review data and opioid prescribing records with patient outcomes to assess provider adherence with opioid prescribing legislation and its association with patient outcomes. To quantify compliance with the statute, the authors examine the days’ supply of opioids prescribed and whether a PDMP check was conducted prior to issuing a second opioid prescription. To evaluate the relationship between provider statutory adherence and patient outcomes, the authors analyzed indicators of chronic or aberrant prescription opioid use and healthcare utilization within 6 months after the initial opioid prescription.

## METHODS

The study was conducted within an academic-affiliated healthcare system in Colorado with over 4 million outpatient visits annually. All medications are electronically prescribed through a shared electronic health record (EHR) system. Throughout the study period, all providers had access to the Colorado PDMP through a single sign-on, EHR-integrated interface, allowing seamless review of patient-specific PDMP data without leaving the EHR or logging into a separate system. Provider engagement with the PDMP was identified through EHR click-level navigation of the integrated PDMP access.

Communication regarding the legislation change followed the standard process of the Colorado Department of Regulatory Agencies. Colorado Department of Regulatory Agencies’ Division of Professionals and Occupations disseminated information through its website and through email notifications sent to all licensed providers in May 2018^18^,^19^ and hosted a question-and-answer webinar and a telephone town hall that included details about the legislation in September 2018. Notably, the healthcare system did not provide additional education or guidance to providers regarding the policy change.

### Study Sample

This retrospective cohort study included adults aged 18–89 years who received an opioid analgesic prescription from a primary care or surgical specialty provider within the system. Eligible patients had a face-to-face visit between May 2018 and May 2019 and were classified as opioid naive, defined as having no opioid prescription in the system’s EHR in the 12 months preceding the index opioid prescription. This definition aligns with the language in SB18-022, “...not had an opioid prescription in the last twelve months by that physician or physician assistant.”^17^ Face-to-face visits were utilized to assure proper data linkage between prescribing and outcome measures. A subset of patients who received a second opioid prescription during the study period was analyzed separately to assess provider adherence to the legislation at the second visit. Exclusions included opioid prescriptions for antitussives, antidiarrheals, intrathecal and epidural drugs, and medications for opioid use disorder. Patients with active cancer diagnoses or orders for palliative or hospice care were also excluded. Legislative exceptions for pain duration could not be assessed in the data set. To ensure accurate linkage between PDMP checks and electronic prescriptions in the same visit, telephone encounters were excluded.

### Measures

The analysis data set was constructed by linking patient-level PDMP and visit-level EHR data. The hospital system collects biological sex at birth regularly but not gender identity, which was excluded from the analysis. Patient-level opioid prescription data were obtained from the Colorado Department of Public Health and Environment (CDPHE), which maintains a research-accessible copy of the PDMP database. Visit-level provider actions and routine patient health data were extracted from the hospital system EHR using automated queries of the system’s centralized health data warehouse (Health Data Compass). To facilitate record linkage, the minimum necessary personally identifiable information from the EHR was securely shared with CDPHE to identify matching PDMP records from 2016 to 2019. Record matching was performed using Link King (Version 7.1), which uses probabilistic and deterministic methods to evaluate the similarities between data elements to determine whether the similarities are strong enough to be considered a record match. Matches range from 100% match Level 1: High certainty through Level 6: Maybe.^20^^,^^21^ For this study, only records with perfect or Levels 1–3 were considered a valid match, resulting in successful linkage in 86% of unique patients. No manual review of matches was conducted.

Matched PDMP data were sent to Health Data Compass, which served as an honest broker responsible for securely merging EHR and PDMP data sets and deidentifying the final data set for analysis. This study was approved by the Colorado Board of Pharmacy, the CDPHE, and the IRB with a waiver of informed consent. A data-use agreement governed the handling and use of the PDMP data by the honest broker and the research team. The final deidentified data set included visit-level patient information, provider PDMP utilization, electronic prescriptions from the EHR, and filled prescriptions in the PDMP.

This study examined provider actions in relation to state legislation and patient outcomes. Adherence to legislation was determined by 2 criteria: (1) the number of days’ supply for both the first and second opioid prescriptions individually and (2) whether the PDMP was accessed during the encounter in which a second prescription was written. Chronic opioid use was defined using PDMP data as filling >90-day supply of opioids within 180 days after the initial prescription.^22^ Aberrant opioid use was defined as filling opioid prescriptions from ≥5 distinct providers or ≥5 different pharmacies within 6 months after the initial opioid prescription using PDMP data.^22^, ^23^, ^24^ To address concerns that limiting the duration of opioid prescriptions may result in inadequate pain management and increased healthcare utilization,^25^ the total number of healthcare system visits for each patient was quantified over the 6 months after the initial opioid prescription.

### Statistical Analysis

The authors conducted a descriptive analysis to quantify the frequencies and percentages of patient characteristics as they related to opioid use as well as prescriber characteristics associated with adherence to statutory requirements for opioid prescribing and PDMP checks. The proportion of prescriptions consistent with the legislation—defined as ≤7 days of opioids—was examined at both the first and second face-to-face prescribing encounter. In addition, the authors calculated the percentage of second encounters in which providers accessed the PDMP before electroniclly prescribing an opioid. Patient outcomes were described by the proportion of individuals with aberrant use and chronic use and by the mean number of healthcare visits, stratified by provider adherence to the statute. Chi-square tests were used to examine the associations between chronic opioid use and (1) receiving >7 days’ supply and (2) whether a prescriber PDMP check occurred. Logistic regression models examined patient outcomes while controlling for key confounders. All analyses were performed using R (Version 4.1.1).

## RESULTS

**Figure 1:**
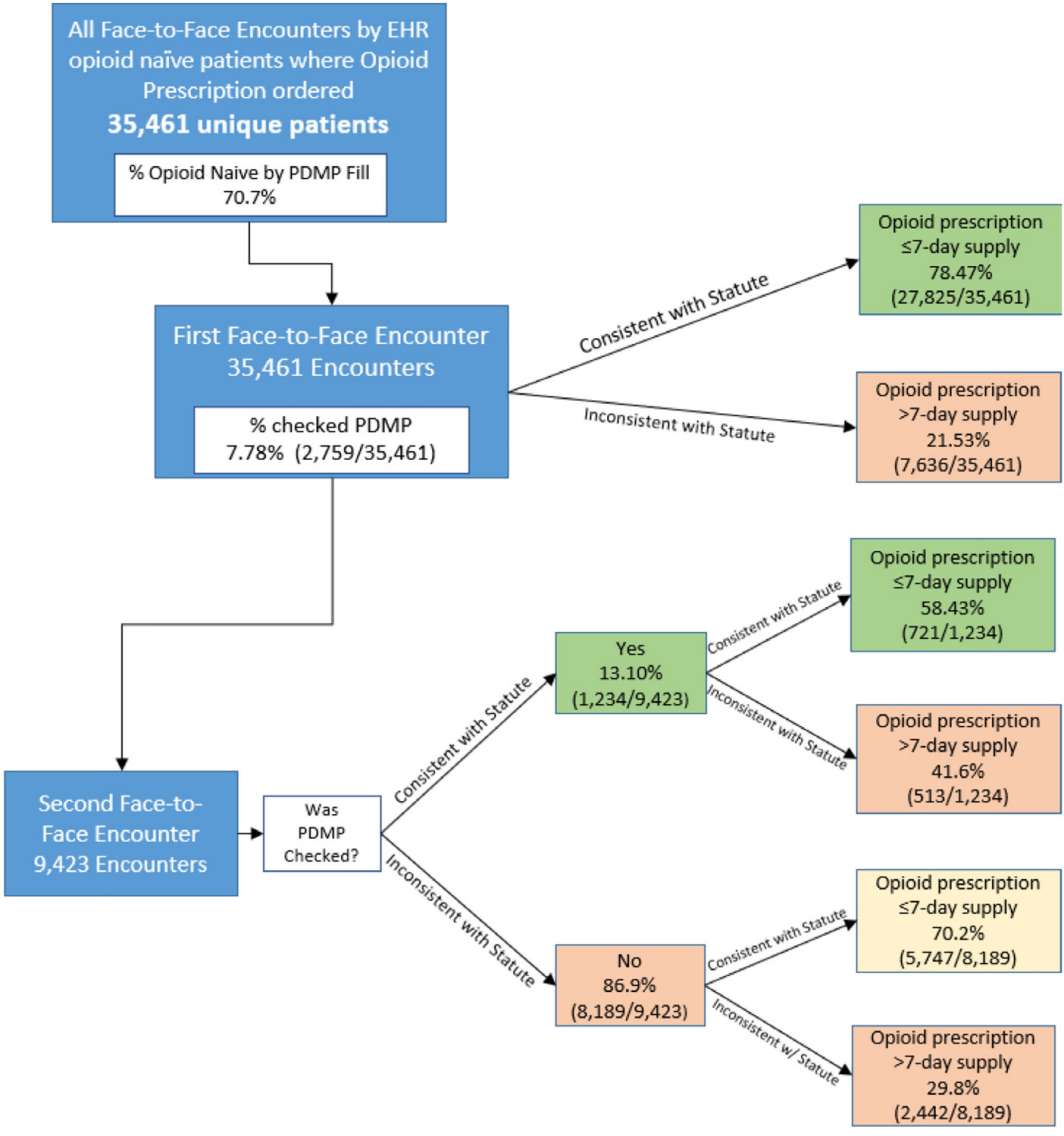
Outcome percentages by provider statute adherence.

*Note*: Aberrant use is defined as opioid prescriptions filled from 5 or more prescribers or 5 or more pharmacies in a 6-month period. Chronic use is defined as filled >90-day supply of opioids within a 180-day timeframe.

Figure 1 presents a flowchart of provider actions and patient outcomes. A total of 35,461 unique opioid-naïve patients received an initial opioid analgesic prescription during the observation period. Of these, 9,423 patients (26.6%) had a subsequent face-to-face visit, during which a second opioid prescription was prescribed. A higher proportion of patients with public insurance received a second prescription. Notably, PDMP data revealed that 30% of patients classified as opioid naïve on the basis of the system’s EHR data had evidence of prior opioid prescriptions, indicating that they were not truly opioid naïve. Table 1 presents the full demographic characteristics of the overall study cohort and the subgroup of patients who received a second prescription.

**Table 1:** Demographics of Opioid-Naïve Patients Eventually Prescribed an Opioid

		First visit		Second visit	
Patient demographics		*n*	% (x̅)	*n*	% (x̅)
Age, years		35,461	(51.3)	9,423	(55.3)
Sex					
Male		20,547	57.9	5,347	56.7
Female		14,914	42.1	4,076	43.3
Race					
American Indian and Alaska Native		131	0.4	47	0.5
Asian		453	1.3	98	1.0
Black or African American		1,664	4.7	487	5.2
Multiple race		835	2.4	210	2.2
Native Hawaiian/Pacific Islander		76	0.2	9	0.1
Other		2,718	7.7	685	7.3
White or Caucasian		28,634	80.7	7,759	82.3
Unknown		950	2.7	128	1.4
Ethnicity					
Hispanic		4,173	11.8	1,075	11.4
Non-Hispanic		30,080	84.8	8,175	86.8
Patient refused		338	1.0	92	1.0
Unknown		870	2.4	81	0.9
Insurance					
Private insurance		17,134	47.7	4,152	44.1
Medicaid		4,584	12.8	1,324	14.1
Medicare		5,348	14.9	1,752	18.6
Other		331	0.9	90	1.0
None		4,251	11.8	1,148	12.1
Unknown		3,813	11.9	957	10.1
PDMP opioid naïve at index visit^a^		25,071	70.7	5,704	60.5
**Provider demographics**^b^					
Provider type					
MD/DO		26,856	76.2	7,149	76.9
NP		1,743	4.9	483	5.2
Other		6,703	18.9	1,666	17.9
Unknown		159	>.01	125	<.01
Clinic type					
Inpatient		16,377	46.2	4,467	47.4
Outpatient		17,742	50.0	4,781	50.7
Other/unknown		1,342	3.8	175	1.9

a Analysis conducted on opioid-naïve patients on the basis of EHR records, yet PDMP records indicate that some patients received opioids from external providers. The methods section provides more details.

b Provider demographics are based on patient record data and, thus, may contain duplicates of the provider/clinic type. DO, doctor of osteopathic medicine; MD, doctor of medicine; NP, nurse practitioner; PDMP, prescription drug monitoring program.

Table 2 presents patient outcome rates in relation to provider adherence with statutory requirements. Providers complied with the statute with an initial opioid prescription of ≤7-days in 78.5% (27,825 of 35,461) of the cases. Among 9,423 second face-to-face encounters with a second opioid prescription, the PDMP was accessed 13.1% (1,234) of the time. For comparison, the PDMP was accessed in 7.8% (2,759) of initial opioid prescribing encounters. Interestingly, when the PDMP was accessed during the second encounter, 58.4% (721 of 1,234) of prescriptions adhered to the 7-day limit, compared with 70.2% (5,747 of 8,189) when the PDMP was not checked.

**Table 2:** Patient Outcomes in 6 Months After Index Visit Stratified by Adherence to Statute

Visit		Day supply of opioids	Total patients *n* (%)	Chronic use *n* (%)	Aberrant use *n* (%)	Follow-up visits Mean (IQR)
First visit *n*=35,461		≤7 days	27,825 (78.5%)	62 (0.2%)	589 (2.1%)	1.95 (1)
		>7 days	7,636 (21.5%)	2,433 (31.9%)	303 (4.0%)	5.44 (6)
Second visit *n*=9,423	PDMP checked, yes	≤7 days	721 (58.4%)	4 (0.6%)	57 (7.9%)	3.25 (3)
		>7 days	513 (41.6%)	275 (53.6%)	32 (6.2%)	8.00 (5)
	PDMP checked, no	≤7 days	5,747 (70.2%)	29 (0.5%)	261 (4.5%)	2.72 (3)
		>7 days	2,442 (29.8%)	932 (38.2%)	136 (5.6%)	6.40 (5)

*Note*: Chronic opioid use is defined as filled >90-day supply of opioids within a 180-day timeframe. Aberrant opioid use is defined as opioid prescriptions filled from 5 or more providers or 5 or more pharmacies in a 6-month period. Follow-up visits are defined as the total number of healthcare visits to the hospital system in the 6 months after the index visit.

PDMP, prescription drug monitoring program.

The prevalence of chronic and aberrant opioid use was evaluated in relation to provider adherence to legislation. Among patients who received a >7-days opioid supply on the initial prescription, chronic opioid use occurred in 32% (2,433 of 7,636), compared with 0.2% (62 of 27,825) of patients who received a ≤7-day supply. Across the entire cohort, chronic opioid use was more likely for patients given >7-day supply (chi-square_1_= 9,210, *p*<0.001) and when the provider checked the PDMP (chi-square_1_=338, *p*<0.001). Among patients receiving a second prescription, chronic use was more common when the second prescription was >7-day supply, regardless of whether the PDMP was accessed (53.6%; 275 of 513) or not (38.2%; 932 of 2,442). Aberrant prescription opioid use was low overall. Among patients whose providers adhered to prescribing limits and checked the PDMP before the second prescription, aberrant use occurred 2.1% (589 of 27,825) of the time after the first and 7.9% (57 of 721) after the second opioid prescription. When a prescription exceeded the recommended 7-day supply, aberrant opioid use remained low with first prescription rates at 4% (303 of 7,636) and second opioid prescription rates at 6.2% (32 of 513) when the PDMP was checked and 5.6% (136 of 2,442) when the PDMP is not checked. Across the full sample, aberrant use was more likely among patients initially prescribed >7-day supply (chi-square_1_=84.3, *p*<0.001) and when the provider checked the PDMP (chi-square_1_=105.3, *p*<0.001).

Patients prescribed an initial 7-day or less supply of opioids had fewer subsequent healthcare visits, with a mean of 1.95 visits (IQR=1), compared with 5.44 mean total visits (IQR=6) when supply was >7 days. Fewer subsequent health visits were also seen for the second opioid prescription. When the provider checked the PDMP, follow-up visit rates were 3.25 mean total visits (IQR=3) with a prescription ≤7 days versus 8 mean total visits (IQR=5) when supplied >7 days. Without a PDMP review, follow-up visit rates were 2.72 mean total visits (IQR=3) with a prescription ≤7 days versus 6.4 mean total visits (IQR=5) when supplied >7 days.

Chronic opioid use was more prevalent when the PDMP was checked prior to the second prescription (22.6%; 279 of 1,234 compared with 11.7%; 961 of 8,189 when it was not). Aberrant opioid use was 7.2% (89 of 1,234) when the PDMP was accessed versus 4.8% (397 of 8,189) when it was not. Logistic regression models of the index opioid visit controlling for patient age, sex, opioid naivety by PDMP, and provider type found that patients for whom the providers checked the PDMP had significantly higher odds of chronic opioid use (OR=2.19; 95% CI=1.94, 2.47; *p*<0.001) and aberrant use (OR=2.12; 95% CI=1.76, 2.54; *p*<0.001) than patients for whom the PDMP was not checked (Table 3). Mean total healthcare visits were 5.6 when prescribers checked the PDMP versus 4.6 mean total visits when it was not checked.

**Table 3:** Adjusted ORs for Predictors of Chronic and Aberrant Opioid Use After Index Opioid Visit

	Chronic use			Aberrant use		
Characteristic	OR	95% CI	*p*-value	OR	95% CI	*p*-value
Age, years	1.02	1.02, 1.02	<0.001	1	1.00, 1.01	0.5
Sex						
Female	—	—		—	—	
Male	0.87	0.80, 0.95	0.002	0.99	0.86, 1.13	0.9
Checked PDMP	2.19	1.94, 2.47	<0.001	2.12	1.76, 2.54	<0.001
Opioid naïve						
Fully naïve	—	—		—	—	
Naïve in system	0.09	0.08, 0.10	<0.001	0.27	0.24, 0.32	<0.001
Provider type						
Primary care	—	—		—	—	
Surgical	0.97	0.89, 1.06	0.5	1.3	1.14, 1.50	<0.001

## DISCUSSION

Using novel methods to link visit-level actions—specifically, opioid prescribing and PDMP review—with patient outcomes, the authors found that although providers largely adhered to legislative limits on opioid days’ supply, they infrequently performed statutorily mandated PDMP reviews. Adherence to the days’ supply limit was associated with lower rates of chronic opioid use and fewer subsequent health visits. However, this association was not observed with PDMP review. Prior evaluations of PDMP interventions have been limited by challenges such as the inability to link provider PDMP review to specific prescriptions or patient outcomes, different data sources, accuracy,^26^ outcomes measured, differences in PDMPs, and variability in state PDMP mandates.^27^ This study addresses these limitations by leveraging EHR click navigation data to reliably capture provider PDMP use and prescribing actions during specific clinical encounters, enabling the connection of visit-level provider actions to patient-centered outcomes.

The selective adoption of one aspect of the legislative mandate—high adherence to days’ supply of opioids but low compliance with PDMP review—is important to consider when evaluating the effectiveness of policy interventions. Although days’ supply limits are straightforward to implement, including PDMP data interpretations into clinical decision making is less obvious and could benefit from more guidance.^28^^,^^29^ Provider inconsistency in PDMP data interpretation is well documented in the literature. Despite the face validity of PDMP checks as a tool to improve prescribing safety, their utility is undermined by variability in provider engagement and understanding.^29^, ^30^, ^31^, ^32^, ^33^ Evaluations of legislation mandating PDMP use prior to opioid prescribing have produced mixed results regarding changing clinical workflows and prescribing behavior. Some studies have reported increased PDMP use,^34^^,^^35^ decreased opioid prescribing,^36^, ^37^, ^38^ and a decrease in days’ supply,^37^ whereas others have found no change in prescribing rates^39^ and an increase in the number of days’ supply.^23^ With respect to patient outcomes, Wen et al.^40^ found that mandated PDMP use was associated with reduced rates of opioid-related inpatient stays and emergency department visits over a 6-year period. In contrast, the evaluation—which did not limit the subsequent visits to those explicitly related to opioids—did not find that PDMP use was associated with a reduction in mean total visits in the 6 months after the initial opioid prescription.

Although prior studies have reported that opioid prescribing legislation did not significantly impact prescribing behavior,^41^, ^42^, ^43^ the findings indicate high provider adherence to Colorado’s statutory limit of ≤7-day supply. This aligns with the statewide downward trend in opioid prescriptions exceeding 7 days, which preceded and was not impacted by the legislation.^44^ This suggests that the observed adherence to days’ supply legislation in this study may reflect ongoing changes rather than the direct effect of the statute. Similar patterns of overall reductions in opioid prescribing independent of legislative changes have been observed in other states.^14^^,^^45^

The finding that chronic opioid use is significantly more common among patients prescribed more than a 7-day supply of opioids is consistent with prior research in opioid-naïve patients.^8^^,^^22^^,^^46^^,^^47^ Prior research has shown that increasing days’ supply, additional prescriptions, and higher opioid doses are associated with long-term opioid use.^46^^,^^48^ In this study, the association between an opioid supply of ≤7-days and fewer follow-up healthcare visits could indicate that limiting initial opioid days’ supply does not lead to increased healthcare utilization, countering concerns about undertreatment of pain. Interestingly, providers who issued a second prescription with the statutory supply limit typically did so without a PDMP check. This may indicate that providers consider short-duration prescriptions to be low risk, reducing the perceived need to review the PDMP, despite legislative mandates. Conversely, when providers did review the PDMP, they may have felt reassured by the PDMP information and more comfortable with a longer duration, potentially explaining the higher day's supply with PDMP review.

Mandating provider PDMP use did not result in widespread provider utilization, consistent with findings by Crawford and colleagues^49^ who reported that PDMP use remained low despite a hospital policy mandating PDMP use. In this study, the relatively higher rates of chronic use, aberrant use, and mean total visits among patients whose provider checked the PDMP before a second opioid prescription suggests that mandated PDMP checks alone may not be sufficient to improve patient outcomes. Alternatively, the difference in outcomes for patients where the PDMP was checked versus not checked could reflect unmeasured differences in patient complexity. In other words, providers checking the PDMP could be a proxy for clinical concern in patients with more complex medical needs, poly pharmacy, or other risk factors.

Prior evaluations of robustness of PDMP legislation—such as a weighted scale of mandated PDMP registration/use, good Samaritan laws, naloxone standing orders—have yielded mixed findings regarding their association with overdose deaths^50^^,^^51^ and opioid utilization.^52^ The study’s findings suggest that mandatory PDMP checks may be less effective in mitigating opioid-related public health issues than policies restricting days’ supply. Clearer legislation coupled with supportive PDMP guidelines and provider education may enhance adoption and effectiveness of the interventions.

Improved collaboration between EHR and PDMP vendors is essential to enhance tracking of provider PDMP use during a visit, enabling more rigorous analysis of causal relationships between PDMP review and patient outcomes. Conducting early and planned evaluations of proposed legislation can facilitate the collection of robust baseline data, allowing for more accurate assessments of policy impacts prior to implementing new legislation. In addition, future research should explore longitudinal trends in PDMP utilization because prior studies have found that prescribing actions aligned with legislative mandates decreases over time despite initial adoption.^18^^,^^19^

### Limitations

This retrospective approach was limited to EHR data collected during routine clinical care. To reliably connect PDMP review documentation with prescriptions at the visit level, the authors only included face-to-face visits, meaning that telephone and video prescribing may have been missed. Patients receiving telehealth services may differ from in-person patients; the authors were unable to assess the extent of these differences. However, the authors do not anticipate differences in provider behavior across visit types. The future healthcare utilization outcome measure was restricted to visits within the authors’ system and did not account for the clinical indication for the prescription/visit. Although patients may have sought healthcare services outside of the authors’ system, the authors expect such utilization differences to be evenly distributed across patient cohorts defined by provider behavior. High healthcare utilization has been used as a proxy for poorly treated pain^53^; however, frequent use of healthcare services could also indicate greater access to health services or more willingness to seek out health interventions. Owing to significant variation in diagnosis codes, the authors were unable to stratify results by diagnosis or indication. Future research may benefit from developing methods to assess reasons for or appropriateness of opioid prescribing. Notably, the Colorado statute only applies to the first and second opioid prescriptions for a patient by a given provider. It is unclear why the language focuses on a single provider versus all medical care, particularly because the PDMP is intended to provide a more comprehensive view of the patient’s prescription history. The evaluation was designed to align with statutory language.

Health system–wide integration of the PDMP into the EHR coincided with enactment of SB18-022. This allowed measurement of visit-level PDMP use, but the timing limited the ability to check baseline PDMP check rates before legislation. The authors only included patients determined to be opioid naïve on the basis of EHR system data to minimize duplication of patients and potential bias as well as to better adhere to the language of the legislation. Patients coming into the hospital system with opioid prescriptions from other providers could be a unique cohort for future study. Although Colorado allows for delegate PDMP access to reduce provider burden,^54^ delegates do not have access to the EHR–PDMP integration. As a result, delegate PDMP access was not measurable and could not be included in the analysis and is expected to be low owing to the ease of provider 1-click access versus delegate access requiring manual sign-on to the external state portal plus transcription of patient information. The use of an honest broker for PDMP data merging was essential to maintain compliance with state confidentiality regulations; however, the deidentification process limited the ability to further assess data or linkage quality.

## CONCLUSIONS

Overall, providers adhered to state legislation by limiting opioid prescriptions to a 7-day supply; however, PDMP utilization remained low despite legislation mandating its use. Notably, chronic opioid use and future healthcare utilization within the following 6 months was lower when prescribed ≤7 days supply, regardless of provider PDMP utilization. In contrast, PDMP review was not associated with future opioid use or healthcare utilization. To accurately evaluate the effectiveness and safety of PDMP policies and use, evaluations should incorporate visit-level data (including PDMP utilization and prescribing) and link to patient outcomes. Legislation that lacks empirical evidence of benefit—or demonstrate unintended harm—should be critically re-evaluated and considered for repeal. Future research is essential to better understand how legislative mechanisms can most effectively promote the safe treatment of pain and improve patient outcomes.
